# A rapid-screening approach to detect and quantify microplastics based on fluorescent tagging with Nile Red

**DOI:** 10.1038/srep44501

**Published:** 2017-03-16

**Authors:** Thomas Maes, Rebecca Jessop, Nikolaus Wellner, Karsten Haupt, Andrew G. Mayes

**Affiliations:** 1 Cefas, Centre for Environment, Fisheries, and Aquaculture Science, Pakefield Road, Lowestoft NR33 0HT, UK; 2 School of Chemistry, University of East Anglia, Norwich Research Park, Norwich, NR4 7TJ, UK; 3 Institute of Food Research, Norwich Research Park, Colney Lane, Norwich, NR4 7UA, UK; 4 Sorbonne Universités, Université de Technologie de Compiègne, CNRS Enzyme and Cell Engineering Laboratory, Rue Roger Couttolenc, CS 60319, 60203 Compiègne Cedex, France

## Abstract

A new approach is presented for analysis of microplastics in environmental samples, based on selective fluorescent staining using Nile Red (NR), followed by density-based extraction and filtration. The dye adsorbs onto plastic surfaces and renders them fluorescent when irradiated with blue light. Fluorescence emission is detected using simple photography through an orange filter. Image-analysis allows fluorescent particles to be identified and counted. Magnified images can be recorded and tiled to cover the whole filter area, allowing particles down to a few micrometres to be detected. The solvatochromic nature of Nile Red also offers the possibility of plastic categorisation based on surface polarity characteristics of identified particles. This article details the development of this staining method and its initial cross-validation by comparison with infrared (IR) microscopy. Microplastics of different sizes could be detected and counted in marine sediment samples. The fluorescence staining identified the same particles as those found by scanning a filter area with IR-microscopy.

Plastic litter, both at the macro and micro scale, is widespread and has accumulated worldwide in the marine environment. Due to ultraviolet (UV) radiation, oxidation and mechanical forces, plastic items break down into increasingly smaller microplastic fragments, below 5 mm in diameter^1^,^2^. Micro-sized fragments such as synthetic fibres from textiles, facial cleansers and many other products also introduce microplastics directly^2^,^3^,^4^. This has led to a build-up of microplastics of varying sizes, composed of different polymer types, across a wide array of marine habitats. Because of their size, microplastics are available and ingested by a broad range of organisms^5^,^6^,^7^,^8^,^9^,^10^,^11^, possibly threatening ecosystems and even human health^12^. The risks that microplastics pose to marine life and humans are widely recognized and have been included in national and international marine protection strategies, policies and legislation (e.g. EU Marine Strategy Framework Directive^13^). Knowledge of plastic concentrations, spatial and temporal changes, sizes, polymer distributions and fragmentation dynamics are a prerequisite for understanding fate and impact of microplastics. To monitor spatial and temporal trends of microplastics, simple, cost-effective and standardized protocols, capable of efficiently and accurately enumerating microplastics in a wide variety of environmental matrices, need to be developed.

Various floatation and density approaches have been described for microplastic studies in sediments^14^. Using the density increase caused by added salt solutions, microplastics float so they can be separated, filtered and analysed. Water column studies can use density separation or direct filtration methods for sample recovery. Biota studies will need to separate microplastics from the surrounding tissues after which they can be processed similar to water or sediment samples^15^. Such approaches lead to many filters containing various materials, including the putative microplastic fragments, which need to be identified and counted. For larger microplastics (0.3 to 5 mm) visual sorting is an accepted approach and one of the most commonly used methods for the identification of microplastics (using type, shape, degradation stage, and colour as criteria), but it still requires expert knowledge and judgement while being rather time consuming. In addition to visual quantification, recent studies have applied chemical and physical characterisation^16^, vibrational spectroscopy^5^,^14^,^17^,^18^,^19^,^20^ or electron microscopy^21^,^22^,^23^ to reduce the risk of false positive/negative misidentification, to determine polymer types and to introduce automated routines^22^. Fourier transform infrared (FTIR) and Raman microspectroscopy have been used to allow polymer identification of particles down to a few μm^24^. There are several recent publications on automating IR-microscopy procedures for microplastic identification^20^,^25^,^26^ to make it less labour intensive, but the techniques are not routinely applied for monitoring, because they are limited by slow speed, high cost and poor spectral resolution, which makes processing of larger sample sets by micro-spectroscopy challenging.

IR microscopy requires technical expertise and assignment of individual particles from their spectral fingerprints is error prone, especially for small particles (<20 μm) where microscope resolution inevitably includes spectral signals from the surroundings (i.e. other adjacent particles or the filter itself). Polymers collected from the marine environment may have been exposed to UV induced photodegradation, thermal degradation and biodegradation, altering the original polymer composition^27^. Bacteria within the coastal and marine environment can rapidly colonize microplastics, forming successional plastisphere-specific bacterial assemblages^28^. These degradation processes and biofilms, in combination with polymer additives, will further complicate spectroscopic analysis due to spectral changes and insufficient reference spectra for polymer degradation products^29^. This problem becomes more apparent for small particles, where the high surface to volume ratio makes the signals from surface material more significant. Many particles will thus fall into an unidentifiable category which is difficult to distinguish from natural polymers such as lignocellulose, chitin etc. Despite these shortcomings, the above-mentioned spectroscopic methods are the methods of choice for most studies of microscopic plastic particles, currently the only available approaches.

To carry out the kinds of spatial and temporal studies necessary for emerging monitoring requirements, as well as addressing new research questions arising from increased awareness of the microplastics problem, much cheaper, faster and more easily applied methods urgently need to be created. Fluorescence staining methods provide a simple and sensitive approach to highlighting specific objects or structures in biological and medical studies. Andrady^30^ proposed the use of a lipophilic fluorescent dye, such as Nile Red (NR) to stain microplastics in surface water samples, facilitating visualisation under a microscope, but this observation has not been followed up to date. NR is a lipid soluble fluorescent dye which allows the *in-situ* staining of lipids. It has been frequently employed to evaluate the lipid content of animal cells and microorganisms, such as mammalian cells, bacteria, yeasts and microalgae^31^,^32^. Furthermore, NR is solvatochromic, so its fluorescence emission spectrum shifts depending on the polarity of its environment. This behaviour might allow microplastics to be categorised into types based on their general hydrophobicity e.g. polyolefin, polyaromatic, polar (polyesters/nylons), or it could provide a useful indicator to evaluate residence time via temporal changes in surface properties due to oxidation or biofouling in the environment. In this manuscript, we present a detailed development and evaluation of this approach for the rapid screening of sediment samples for microplastics.

## Results

Multiple dyes (Oil red EGN, Eosin B, Rose Bengal, Hostasol Yellow 3G and NR) were tested for their ability to adsorb to plastics. NR was adopted, since it was the most effective in terms of adsorption and fluorescence intensity. The optimum dye concentration (between 1 and 1000 μg mL^−1^) and incubation time (between 5 minutes and 66 hours) for visibility was determined. Using higher dye concentrations increased the fluorescence intensity of the dyed particles, but also increased the background signal from the Whatman filters. A working concentration of 10 μg mL^−1^ gave a good balance between visibility, speed and background signal. Fluorescence intensity increased rapidly with incubation time, but plateaued after 30 to 60 minutes and remained constant up to 66 hours. Incubation times longer than 30 to 60 minutes led to gradual aggregation of the unadsorbed dye (which has low water solubility) and stronger colouring of the filters, especially in the presence of higher concentrations of zinc chloride used to increase density. For most studies, incubation with 10 μg mL^−1^ NR for 30 minutes was adopted for staining.

Different concentrations of ZnCl_2_ (from 0 to 1.8 g/g water) were trialled to determine the best density to cause microplastics to float, while ensuring that the vast majority of inorganic mineral particles and other potential interfering material sedimented during centrifugation^23^. A density of 1.37 g mL^−1^ provided a good compromise between maximising recovery and minimising interference from excessive unwanted particulates. Most common plastics have a density well below this value^33^, while it is close to the density of PVC and PET (an important subset of frequently observed marine microplastics), hence only a very few unusual plastics (e.g. fluoropolymers) or dense composites would potentially be removed by sedimentation. Crab claw fragments, which showed a dull orange/red fluorescence, might give false positives in the counting. However, they are heavily mineralised with calcium carbonate, have higher density than plastics and are sedimented under the conditions of extraction (Fig. SI6).

**Figure 1:**
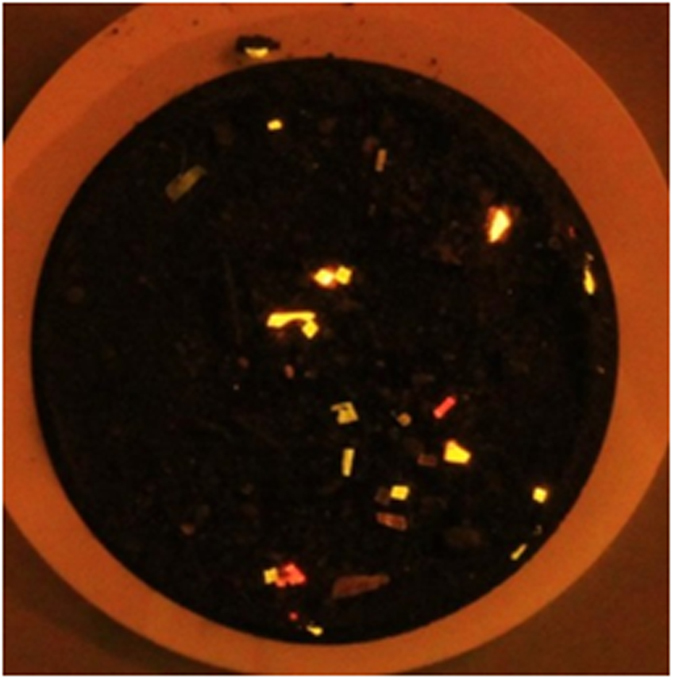
1 g of marine sediment (SPI 6) spiked with microplastics of six different polymer types, dyed with Nile Red (1000 μg mL^−1^, 30 minutes), then filtered onto a 47 mm diameter membrane filter. Photograph taken with a blue light (Crime Lite: 450–510 nm) and orange filter (529 nm).

Results of staining spiked particles of various polymer types in coarse and fine marine sediments (30 particles in each sample) are shown in Table 1, with an image in Fig. 1. The plastic particles fluoresced and could be counted easily (>100 μm). On average a 96.6% recovery rate was obtained. Samples with >100% recovery may have had additional microplastics present from the original sediment. This was confirmed from three unseeded control samples for each sediment. Control samples contained some very small fluorescent “dots”, but also on average about 2 larger fragments per 1 g sediment. This represents microplastic in the control sample and/or a degree of contamination from labware and solutions, since at this stage no precautions were taken to avoid such contamination. This was addressed later by washing all equipment with filtered water (0.22 μm) and pre-filtration of all solutions through 0.22 μm filters (Whatman cellulose nitrate membrane filters or PTFE syringe filters) prior to use. A moistened wooden cocktail stick was used to collect any fluorescent fragments from the samples. Analysis of some of the small fluorescent “dots” from the control sediment by Raman microscopy gave strong bands indicative of calcium carbonate (see Fig. SI7), but the fluorescence staining suggested they were organic and hydrophobic in nature. These were most likely small fragments of mineralised chitin, which could potentially cause false positives. Chitin fragments are not buoyant under the conditions used for actual sample processing (see above) so they are separated from the microplastics and are unlikely to cause significant problems when using the proposed method, which gave very high (>97%) recovery in coarse sand, but a lower recovery of 85–88% in fine silt. This is probably due to a degree of entrapment and burial of microplastics and should be taken into account when reporting microplastic loadings.

From these initial tests, it was also apparent that the different types of plastic displayed different fluorescent colours when stained with NR (Fig. 1). NR is solvatochromic and its fluorescence emission spectrum red-shifts markedly as the polarity of the solvent increases (see Figs SI9–SI11 for spectra and images demonstrating this).

**Figure 2:**
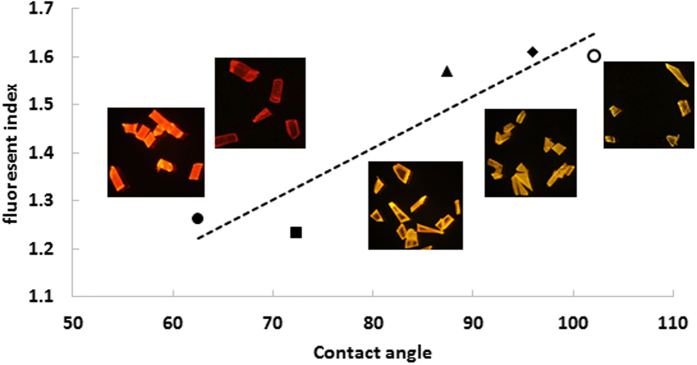
Fluorescent index, represented by (R+G)/R, plotted against published static contact angle values (a measure of the surface polarity). The actual images are inset to show the clear colour variations.

To investigate the potential application of this solvatochromic response, particles of individual known plastics were stained and imaged. The images were processed using Image J to determine the average RGB intensities from the image areas containing the stained plastic fragments. From the values, a simple “fluorescence index” was calculated as (R+G)/R. This equation normalised the overall intensity of the fluorescence and maximised the differences in colour, producing a single value that could be used to represent the “polarity” of the polymer surface. These values are plotted against literature values for static contact angle measurements (an easily-measured proxy for polarity) for these polymers in Fig. 2, where the images of the actual colours observed are inset for reference. The graph shows a clear trend, confirming the relationship between polymer surface polarity and NR fluorescent colour. It was possible to group the polymers into “polar” (nylon, PET) and “hydrophobic” (PE, PP, PS) and this might be a useful distinction for general particle counting and categorisation. Identification of individual polymer types using this approach is unlikely, but it offers promise (with further validation) for general particle categorisation, which might be useful for comparing proportions of different types of plastics with production or usage data to determine behaviour, fate, degradation etc. of plastics in the marine environment. Alternatively, it might provide an interesting tool to assess surface oxidation or biofilm adsorption onto plastic particles in relation to exposure time and conditions, in order to understand better the temporal changes that take place to particle surface properties.

**Figure 3:**
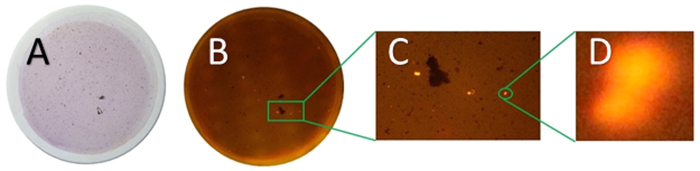
Filter images from processed sediment sample number 805. (**A**) white light, showing a variety of extracted debris; (**B**) Autostitch reconstruction of the 54 tiled images taken using a blue light and orange filter, (**C**) expansion showing three bright spots of fluorescently-tagged microplastics and (**D**) close-up of one larger particle, approximately 100 μm across. A number of bright spots much smaller than this are also clearly visible in image (**C**).

When marine sediment samples were processed using the density extraction procedure, a certain amount of debris (organic material, black carbon fragments, small mineral grains etc.) usually floated to the top of the tubes, along with any microplastic fragments. The amounts and texture of this debris varied greatly depending on the nature and source of the sediment. A typical filter is shown in Fig. 3 (sample 805).

The white light image shows numerous particles on the filter surface, but the reconstructed fluorescence image of the whole filter demonstrates that only a few larger fluorescent particles are present in this sample. To detect smaller particles, it is necessary to zoom in and analyse the filter tile by tile. For method development, a 9 × 6 array of images was used, each one covering approximately 8 mm × 5.4 mm of the filter area, collected using the automated rig (see SI section 1 for details). A single pixel of the 5148 by 3456 pixel image array at this magnification thus represents about 1.5 μm, making it theoretically possible to image particles down to about 5 μm (assuming adequate optical resolution and taking at least 9 connected pixels to represent a real bright object, rather than random noise). Potentially, even smaller particles could be addressed, at the cost of time and effort, by zooming in further and using more tiles to cover the filter. Alternatively, for routine screening, a 7 × 5 array significantly reduces the number of images with little real decrease in the size limit of detection and this has now been adopted for our routine work.

**Figure 4:**
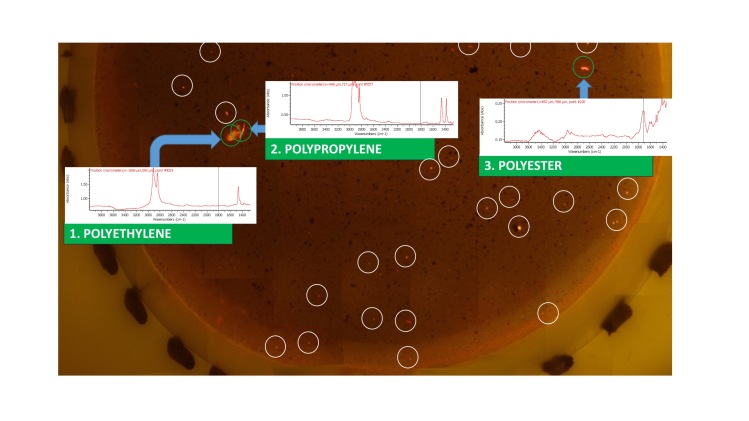
Part of a filter image from sample 805, reconstructed from individual tiles, showing fluorescent particles and, superimposed, the IR spectra obtained by picking the three larger particles and transferring them to an Anopore filter. This allowed them to be identified as common microplastics. Note also, the many additional small bright particles (25 have been ringed for clarity), which were too small to transfer reliably.

**Figure 5:**
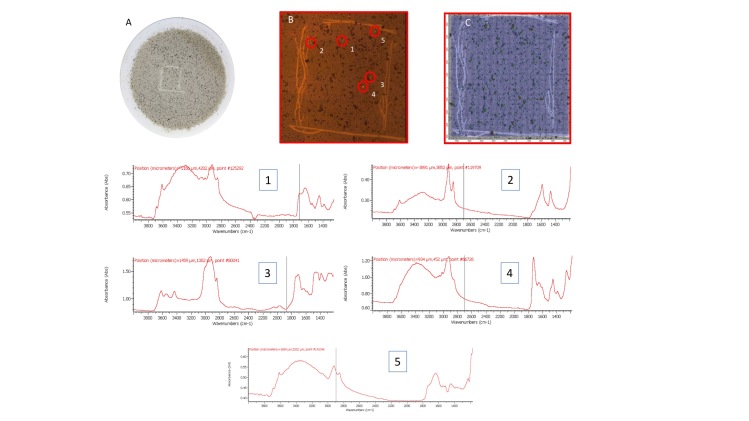
Image of the filter in white light showing (**A**) the scribed area; (**B**) expansion of the scribed area under blue light, photographed through an orange filter, reconstructed from tiled images showing the bright fluorescent objects identified, (**C**) tiled white-light image from the IR microscope overlaid with a C-H filtered IR spectral map to highlight organic material and below, IR spectra from the 5 locations ringed and numbered in panel (**B**).

A typical result from a tiled filter image is shown in Fig. 4, where part of a filter is shown, reconstructed from its individual tiles using free software Autostitch^34^. In Fig. 4, three larger fluorescent particles were observed. These were sampled with a moistened cocktail stick and transferred to a clean Anopore filter for analysis by infrared microscopy. The corresponding IR spectra are superimposed. This allowed the microplastics to be identified as polyethylene, polypropylene (fibre) and polyester (fibre) respectively. (More details from the IR microscope are shown in ESI Figs 12–14). Careful analysis of this filter image, however, also shows at least an additional 25 small bright spots, which are also putative microplastics. These were too small to pick up and transfer reliably, however, so for the very small fragments an alternative approach was taken to validate the fluorescence staining result and demonstrate that these small fragments are indeed microplastics. A sample of sediment 295 was extracted using our method and filtered directly onto a 47 mm Anopore filter. This was observed and photographed under white and blue light and an area where a few very small bright spots could be seen was identified. An approximate 1 cm square was marked in the filter surface by scratching with a metal point, then the filter was fluorescence-imaged using 35 tiles in the normal way. The scribed area was reconstructed from the images. The filter was then transferred to the IR microscope and the whole scribed square scanned in rapid-scan mode. The various stages of this experiment are depicted in Fig. 5 (with larger versions of the spectra available in ESI Figs 15–20). The IR data were filtered for C-H stretch signals between 2800 and 3000 cm^−1^ to identify any organic material. Many particles were highlighted (Fig. 5c), but inspection of the spectra at most of these locations (>100 were checked) indicated a consistent fingerprint of partially-oxidised carbonaceous material, which did not correspond with any common plastic. This is most likely “black carbon” material arising from decay of organic matter and it is clear from the fluorescence image that this material was not labelled with NR. Five locations were identified, however, with significantly different spectra.

Spectra from highlighted locations 1–5 emphasise the problem of accurately identifying small plastic particles. These areas correspond with bright spots on the fluorescence image. Four notably different spectra are present and undoubtedly originate from polymeric material, but none can be assigned with complete confidence. Significant signals are present in the OH/NH stretch region between 3000 and 3700 cm^−1^, but these are weaker relative to the C-H stretches than would typically be seen for natural carbohydrate based polymers such as cellulose, carrageenan or chitin, or for proteinaceous material, suggesting that they are indeed anthropogenic. Particle 3, identified in Fig. 5, has characteristic features of PET, in particular, the signals around 3500–3700 cm^−1^ and 1970 cm^−1^, as well as the strong carbonyl signal at 1730 cm^−1^. There are also notable differences between the spectra in the 1400–1800 cm^−1^ region, which indicate that particle 2 (Fig. 5) may be a polyamide, but particles 1 and 4 both have (different) balances of amide-like and ester character, which are difficult to characterise with confidence. This most likely results from heavy weathering and/or biofouling, introducing a complex balance of chemical functionality into the spectrum. Uncertainties over precise assignment notwithstanding, it appears that the fluorescent particles 1–5 identified by the staining method are indeed microplastic particles, providing validation that the method is robust and accurate in identifying microplastics. Inspection of the IR spectra around location 5 identified a single spectrum that had a form similar to particle 1. Since a 25 μm aperture was used in the IR spectral imaging, this indicates that the particle must be very small, despite the quite bright spot on the fluorescence image. This indicates that even small microplastics are being picked up by the method.

The possibility that algae might stain using NR and hence produce false-positives in the method was an important consideration due to their prevalence in the marine environment. No fluorescence was observed for any of the three algae cultures tested for interference using the lighting and optics used for microplastic identification (see Figs SI20–22). NR staining of oil droplets in Tetraselmis has been widely reported, however, so this observation was explored further. Imaging with a fluorescence microscope (see Fig. SI20) showed that the algal cells were indeed stained, but high excitation intensity and long integration times for imaging were required, compared with those needed for microplastic fragments under the same microscopic imaging conditions.

## Discussion

This fluorescence staining method, in combination with density separation, provides a simple and sensitive approach to highlighting most common polymer fragments in marine sediments. The plastic types used in this study cover roughly 75% of annual European plastics demand and hence represent the majority of plastic fragments likely to be found.

Validation required a demonstration that common materials and structures likely to be present in marine samples did not give false positives. From algal staining studies, we showed that while some algae may indeed be stained by NR, our protocol is quite inefficient for algal staining, lacking the higher levels of organic solvent usually used to enhance dye penetration into the algae, hence their fluorescence is weak in comparison to polymer particles, and they are not observed when imaged on the filter analysis rig. Similarly, other organic detritus, such as seaweeds, wood, feathers and various types of mollusc shells were shown to stain either very weakly or not at all, suggesting that the method has good selectivity for plastics under the conditions applied. Further discussion can be found in the ESI.

The preliminary results showed that the solvatochromic behaviour of NR generated distinctively different colours for fragments from different types of polymers. This allowed microplastics to be grouped by polymer polarity and offers the potential to do basic polymer typing in the future. There is a need to further validate this “colour typing”, however, to assess more fully the effects of intrinsic plastic colouration, weathering and biofouling. Indeed, it may provide a simple and effective tool for following these processes during environmental exposure, so further exploration of this behaviour would be valuable. While we have only tested a selection of polymers, they represent a wide range of polarity and surface functionality. Given the mode of interaction of NR with polymer surfaces (mainly van der Waals interaction with additional dipole interactions in some cases) there is no reason to suppose that it would not adsorb to any given polymer surface, including hard plastics, rubbers, resins etc.

Microplastics of different size fractions were observed in marine sediment samples using the described method and subsequently validated by FTIR microscopy. As a result of the fluorescent staining, microplastic fragments of a range of sizes and polymer types became clearly visible in blue light, which allowed them to be differentiated from other debris, making it much easier to sort samples and assess microplastic abundance. The results showed fluorescing microplastics on the filters, with sizes from several hundred μm down to a few μm. The observed fluorescent particles in marine sediments indicates that microplastics have been settling down from the water column. Microplastics in the low μm range have rarely been reported, due to analytical issues and/or detection limits^35^. Our preliminary results indicate that microplastic abundance in sediments might have been underestimated previously. Further details of this work will be published elsewhere.

Depending on the required accuracy/certainty of analysis, the technique presented here can be used as a standalone technique for microplastic counting or in combination with existing FTIR or Raman instrumentation to speed up the process of object selection. The very small amounts of NR adsorbed on the particles did not interfere with IR or Raman spectroscopy. For instance, the white-light imaging optics in a FT-IR microscope could be easily adapted to excite with a blue LED and image through an orange filter to provide a fluorescence picture of a filter area, which could guide the operator directly to the microplastic fragments for IR imaging. As a stand-alone technique, the basic staining method allows for the detection and counting of particles down to a few microns using the described methodology, making it easy and inexpensive to apply globally in laboratories with basic equipment while providing a minimum standard operating procedure for microplastic quantification.

Very small objects down to a few micrometres could be detected on images of higher quality and thus the size limit of detection is defined by magnification and optical resolution. Already at this stage, sufficient microplastics were detected to complicate visual counting. Further improvements to the visual analysis are currently being developed, with automated image recognition/counting/measurement and RGB characterisation algorithms based on the polarity index. Additional future developments are also envisaged by combining this approach with other image-based analytical methods to allow identification of the individual types of plastic. This would provide an even more powerful analytical approach, though the current method as described provides a simple and effective staining method to visualise microplastics. With appropriate alterations to the protocol, filtration steps to reduce volumes for water samples or digestion/solvent extraction methods generally applied for biota, the method should also be applicable to other matrices in which microplastic analysis is desirable, lowering cost and speeding up quantification processes.

## Methods

### Materials and instrumentation

NR and acetone (AR) were purchased from Sigma Aldrich (Gillingham, UK). Zinc chloride (Acros, SLR) was purchased from Fisher Scientific (Loughborough, UK). Water used was 18 MΩ analytical grade. Whatman 25 mm and 47 mm diameter cellulose ester (0.22 μm), cellulose nitrate (0.45 μm) and Anopore (0.22 μm, aluminium oxide) filters were supplied by GE Healthcare. Glass membrane filtration apparati were used for all filtration operations, aided by vacuum from a KNF laboport pump. Photographs were recorded with a Canon EOS 600 or EOS 1200 digital SLR camera. For excitation, a high powered blue LED light source was used (Crimelite 450–510 nm, Foster and Freeman, Evesham, Worcestershire U.K). Fluorescent images were recorded through an orange filter (Kobo or Foster and Freeman, 529 nm) to exclude the incident blue light. FT-IR reference spectra and spectra to identify beach-found plastic litter were recorded on a Perkin Elmer Spectrum BX with a SensIR single pass diamond ATR attachment (16 scans; 4 cm^−1^ resolution). Infrared microscopy was carried out on a Thermo Scientific, Nicolet iN10MX infrared imaging microscope using a variety of settings and imaging modes. Raman spectra were recorded on a WiTec confocal Raman system with 532 nm laser excitation. Individual particles were dried onto gold-coated glass substrates for measurement and laser power was adjusted manually to give the best quality spectra. Fluorescence microscopy was done using a Zeiss SteREO Lumar V12 system comprising Axiocam camera, 2× ILL2500 LCD and an EXFO X-cite series 120. The microscope was fitted with an 80 mm NeoLumar Lens. Samples were placed on clean glass slides, covered with glass coverslips and imaged using transmitted light. Settings for GFP were used, with the installed GFP filter set.

Samples were centrifuged in a Heraeus Biofuge Primo centrifuge with 6 × 50 mL rotor, using disposable plastic 50 mL centrifuge tubes (polypropylene tubes with blue polyethylene screw caps, supplied by Fisher Scientific). Marine sediment samples (supplied by Cefas) were collected from various locations around the UK coast. Each sample was dried in a vacuum oven to constant weight, using a bleed of filtered air to remove moisture from the oven while avoiding contamination from ambient dust. The samples used in this study are shown in Table 2:

### Filter Imaging

The automated filter-scanning rig used a commercial micro-milling machine (Sanven, China) to provide automated XYZ motion, combined with a trinocular microscope head and a photo-adaptor to connect the Canon EOS camera. Further details can be found in the Supporting Information (section 1). The camera was operated via USB using the Canon remote shooting software. The camera was first focused using white light and manually changing the Z-axis of the milling machine. The blue light was then used for fluorescent imaging. A G-code routine was written to control X-Y scanning (listed in ESI section 8) and a series of slightly overlapping photographs were taken to cover the whole filter area. The demonstration version of AutoStitch^33^ was used to generate panoramic image stitching by automatically recognising matching images.

### General method development

Nile Red (NR) stock solution was prepared at 1 mg mL^−1^ in acetone and filtered using a 0.22 μm PTFE syringe filter into a clean glass screw-top vial and used for all the staining experiments. Zinc chloride solutions were made up in analytical water at varying concentrations and filtered through 0.22 μm cellulose nitrate filters into clean glass storage flasks with ground glass stoppers. Analytical water was filtered in the same way and used for suspension of microplastic samples and sediments.

Microplastic fragments (typically 0.1–0.5 mm) were prepared using a sharp scalpel to scrape fragments from blocks of virgin plastic, consumer plastic items identified through their recycling symbols or waste plastics picked from the tide-line on Lowestoft beach. U.K. The identity of all test materials was confirmed by FT-IR measurement prior to use. The plastics used were polyethylene (PE), polypropylene (PP), polystyrene (PS), polyethylene terephthalate (polyester – PET), polyvinylchloride (PVC) and polyamide (nylon 6).

Staining was carried out by adding NR stock solution in acetone to give a final concentration of 1, 10 or 100 μg mL^−1^ in the suspension of microplastics or sediment, with or without zinc chloride, depending on the experiment. Adsorption time varied between 5 minutes and 66 hours in the optimisation study, at varying concentrations. For most work, 10 μg mL^−1^ and an exposure time of 30 minutes was used.

### Method validation

#### Specificity in relation to polymer type

For initial spiking experiments, 1 g of dried sediment was weighed and spiked with a known number of microplastic fragments of six different polymers: nylon, PS, PVC, PET, PE, PP. The sediment was suspended in 5 mL water, dyed with 50 μL NR stock and incubated on a Heidolph Rotamix shaker at 100 rpm for 60 minutes. The sediment was then vacuum filtered (Whatman 47 mm cellulose nitrate filter membrane 0.22 μm). The samples were viewed under a blue light (Crime Lite: 450–510 nm) through an orange filter (529 nm) and seeded microplastics were counted. The filters were also photographed.

To investigate solvatochromism of the adsorbed NR, images containing nylon, PS, PVC, PET, PE and PP fragments were analysed. The fluorescent particles were identified in the images and their RGB values extracted using Image J (https://imagej.nih.gov/ij/). These values were then transformed into a “fluorescent index” value − (R+G)/R – using the 8-bit colour intensity values in the red (R) and green (G) channels to provide a simple comparison value, which can be related to the polymer type.

#### Density separation for microplastic extraction from sediments

For microplastic separation, zinc chloride solutions with differing densities were prepared gravimetrically from a freshly opened bottle of zinc chloride and the densities measured by weighing a fixed (100 mL) volume. Values are given in Table SI1. Microplastics of different known composition were tested for floatation in the various solutions under centrifugal conditions, along with samples of different types of coarse and fine marine sediment. A density of about 1.35 allowed floatation of all the polymer types tested (along with small amounts of sediment material), while the vast majority of the sediment material settled to the bottom of the centrifuge tube. Some sediment material remained buoyant under these conditions. The density could be reduced to decrease the fraction of floating material, but with the risk that some denser microplastics might be missed due to sedimentation.

#### Recovery of spiked microplastics

To test the recovery rate of seeded microplastics from marine sediments, a fine sediment, LIT 81C and a coarse sediment LIT 79C were chosen. Triplicates (3 × 5 g) for each sediment type were weighed and seeded with 20 nylon and 20 PE NR-dyed microplastics. The seeded sediments were slowly added to the zinc chloride solution (30 mL, 1.37 g mL-1), mixed, then centrifuged at 3900 g for 5 minutes with a braking speed of 9. The fluorescent microplastics were collected from the top of the solution with glass Pasteur pipettes using blue/green incident light (450–510 nm) through an orange mask (529 nm) to visualise them. The samples were then made back up to volume with zinc chloride solution and resuspended, then centrifuged and extracted again. This was repeated to give a total of three extractions for each sample. The recovered particles were combined, filtered, photographed and counted.

#### Cross validation and confirmation with FT-IR

For validation of the fluorescent method compared with imaging FT-IR, real unspiked sediment samples were processed as above, then filtered onto 47 mm Anopore filters (0.22 μm; aluminium oxide). Large fluorescing fragments were handpicked using a wooden cocktail stick moistened with ethanol, resuspended in approximately 1 mL ethanol, and filtered onto a small Anopore filter (25 mm) and analysed by FT-IR microscopy in transmission mode, using bare filter to set the background.

Further analysis of even smaller fragments on the 47 mm Anopore filter was carried out by marking an area of about 1 cm by 1 cm. This area contained fluorescing particles as observed under blue/green incident light (450–510 nm) through an orange mask (529 nm). The filter was then photographed using the automated fluorescence scanning rig and the scratched area identified and reconstructed from tiled images. The same area was imaged using the FT-IR microscope in transmission mode, using bare Anopore filter as the background (25 μm × 25 μm pixel size, 1 scan at 16 cm^−1^ resolution)). Putative microplastic particles were identified by filtering the spectral data array for C-H stretch signals around 2800–3000 cm^−1^ (indicating likely organic material). Regions containing significant C-H signals were further analysed to provide spectra for identification. The IR map of the imaged area was compared with the fluorescence image to check for coincidence of the fluorescent particles and the microplastic fragments identified from the IR.

#### Specificity/selectivity in relation to biological materials

A possible drawback of this staining approach is the possibility that false positives might be introduced because of staining biological organisms such as marine algae. These can be found in a wide range of sizes and forms. It is well known that some (though not all) of these organisms can be stained with NR^31^, and indeed this has been developed as a screening assay for algae that produce lipid droplets^32^, due to the interest in this area for biofuel production. In general, algal staining protocols include a water-miscible organic solvent (typically acetone, DMF or DMSO) to improve dye penetration into the organism. Our plastic staining method has a low solvent concentration (1% acetone, introduced from the NR stock, compared with 25% DMSO in an optimised algal staining method) so it is rather inefficient at staining algae. The protocol was tested on three marine algae representing different classes, morphologies and size scales – *Diacronema lutheri* (4–6 μm), *Tetraselmis suecica* (10–15 μm) and *Skeletonema sp.* (filamentous, diameter 2–20 μm). Once stained, the samples were filtered and imaged as for microplastics. Samples of dyed algae and microplastics were also investigated using a Zeiss fluorescence microscope with GFP filter set and settings optimised for green fluorescent protein analysis. A wide range of other organic materials that might be found in sediments (wood, seaweeds, common whelk egg cases, feathers, cotton fibres, paper, crushed shells, crab and shrimp claws etc.) were also tested for NR staining.

## Additional Information

**How to cite this article:** Maes, T. *et al*. A rapid-screening approach to detect and quantify microplastics based on fluorescent tagging with Nile Red. *Sci. Rep.* **7**, 44501; doi: 10.1038/srep44501 (2017).

**Publisher's note:** Springer Nature remains neutral with regard to jurisdictional claims in published maps and institutional affiliations.

## Supplementary Material

Supporting Information

## Figures and Tables

**Table 1:** Recovery of seeded microplastics from sediment samples by direct counting of NR-stained fragments after NR staining with or without inclusion of the density separation step.

Protocol	No extraction step		With extraction	
Matrix amount	0.5 g	1.0 g	5.0 g	5.0 g
Microplastic type	Mixed polymers	Mixed polymers	nylon	PE
Number seeded	30	30	20	20
**Sample**	**CAP1 coarse sand**		**LIT 7C coarse sand**	
Replicate 1	32	27	20	17
Replicate 2	29	27	20	21
Replicate 3	31	30	19	20
Mean	31	28	20	19
S.D.	1.5	1.7	0.5	1.7
Recovery %	102	93	98	97
**Sample**	**SPI 6 fine silt**		**LIT 81C fine silt**	
Replicate 1	28	28	17	16
Replicate 2	29	32	20	20
Replicate 3	30	28	14	17
Mean	29	29	17	18
S.D.	10	2.3	2.4	1.7
Recovery %	97	98	85	88

The mixed polymer sample contained a total of 30 microplastics, 5 each of: nylon, PS, PVC, PET, PE and PP.

**Table 2:**
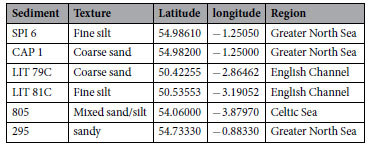
The samples used in this study
