# Supplementary material for: A rapid-screening approach to detect and quantify microplastics based on fluorescent tagging with Nile Red
Source: Sci Rep. 2017 Mar 16;7:44501. doi: 10.1038/srep44501 (PMC5353725; doi:10.1038/srep44501)
Supplement: Supporting Information [file srep44501-s1.pdf]

A rapid-screening approach to detect and quantify microplastics based on fluorescent tagging with Nile Red.

Thomas Maes[1], Rebecca Jessop[2+], Nikolaus Wellner[3], Karsten Haupt[4] and Andrew G. Mayes*[2]

[1] CEFAS, Centre for Environment, Fisheries, Aquaculture and Science, Pakefield Road, Lowestoft NR33 0HT, UK.

[2] School of Chemistry, University of East Anglia, Norwich Research Park, Norwich, NR4 7TJ, U.K.

[3] Institute of Food Research, Norwich Research Park, Colney Lane, Norwich, NR4 7UA

[4] Sorbonne Universités, Université de Technologie de Compiègne, CNRS Enzyme and Cell Engineering Laboratory, Rue Roger Couttolenc, CS 60319, 60203 Compiègne Cedex, France

+ present address: Ricardo-AEA Ltd. Gemini Building, Harwell, Didcot, OX11 0QR, U.K.

Supporting information

1. details of components and construction of the imaging rig
2. table of densities of zinc chloride solutions
3. photograph of centrifuge tube showing sedimentation of chitin and floatation of microplastics
4. Raman spectra of small fluorescent dots extracted from marine sediments WITHOUT density floatation step.
5. Fluorescence spectra of Nile Red in solvents of different polarity and photographs of polymers of similar chemical structure to the solvents showing the correspondence of the solvatochromic shift.
6. Enlarged IR spectra from the FT-IR microscopy
7. Fluorescence images of algae and microplastics stained with NR and comments about other possible false positives.
8. Example g-code listing for X-Y motion of milling machine

1 Design and construction of the automated imaging rig

This was based on a Sanven CNC3020T three-axis micro-milling/engraving machine. Similar systems are available from many online retailers and it is a very cost-effective system for automated x,y,z motion control with reasonable positional accuracy (claimed repeatability of 10 µm). Our system was shipped with a test version of Mach 3 machine control software, but a full version was purchased from Arcsoft to run the instrument.

The mill was modified by removing the milling spindle motor (cylindrical) and replacing it in the clamp with an aluminium cylinder of the same diameter. At the top of this, two studs and wing-nuts were inserted to allow a PVC frame to be clamped in place. The frame was machined to clamp the head of a Motic trinocular microscope firmly in place. This is best explained by reference to photograph SI2. A commercial photomicroscope adaptor was used to connect the imaging port of the trinocular head to the Canon EOS 600D camera. The eyepieces were blanked off with black caps to minimise stray light.

Note: While this optical arrangement works effectively and provides a useful zoom range for this work, a lighter and more elegant solution would be to use an appropriate microscopic imaging lens connected directly to the camera and adapting the mounting arrangements appropriately. This would be smaller, lighter and easier to work with and align. Our system was built as described due to the availability of the parts.

An aluminium holder was machined to hold 47 mm diameter filters in place, with a thin ring machined to weight down the filter around the edges and ensure it is held flat for imaging, while not obscuring any of the actual sample area (the edge of the filter is clamped under the glass flange of the filtration apparatus, so the outermost 3-5 mm of the filter is blank). This holder would probably be better sprayed matt black to avoid any stray reflections, but for fluorescent imaging it works well as it is.

X-y motion was controlled by a G-code script, which moved the camera in a back and forth rectangular grid motion to scan the entire filter area using either a 7 X 5 or a 9 X 6 array. This is a good compromise between maximum speed of scanning and ease of image reconstruction. Staggered arrays could reduce the number of tiles required to cover the circular area, but would potentially be more complex to reassemble. The speed of the scanning motion and the acceleration/deceleration ramps can be adjusted in Mach 3 software to minimise jerking and vibration. This was adjusted by trial and error until a good compromise was found. A delay was also programmed before each image was acquired to allow for any vibrations to be damped, so that images were sharp and focused. The microscope zoom was set to 1 for the 7 X 5 array, but could then be zoomed (up to 4 X for this microscope head) to higher magnification if required.

The rectangular photographic image was set square to the x-y motion by aligning the edge of the field of view with one of the T-slots on the milling machine base by rotating the camera adaptor in the 3rd port of the microscope head.

Images were acquired using the Canon EOS remote shooting software run on a different PC (it is recommended that Mach 3 is run on a dedicated computer, to prevent

unexpected interrupts from disrupting the timing of G-code events). Focus was set by manual adjustment of the z-axis of the mill using the manual movement controls of the Mach 3 software, using white light to illuminate the filter. Once focused, the orange filter was set in place at the base of the microscope head and the sample filter illuminated with blue light from the Crimelite, which was clamped in place to give a uniform lighting across the filter. Exposure for fluorescent imaging was set manually by trial and error prior to imaging the whole filter.

Images were acquired manually, after each movement of the head, by activating the remote shutter button in the software. This action could almost certainly be automated by programming a suitable contact closure signal to the remote shutter input of the camera, but this has not yet been implemented.

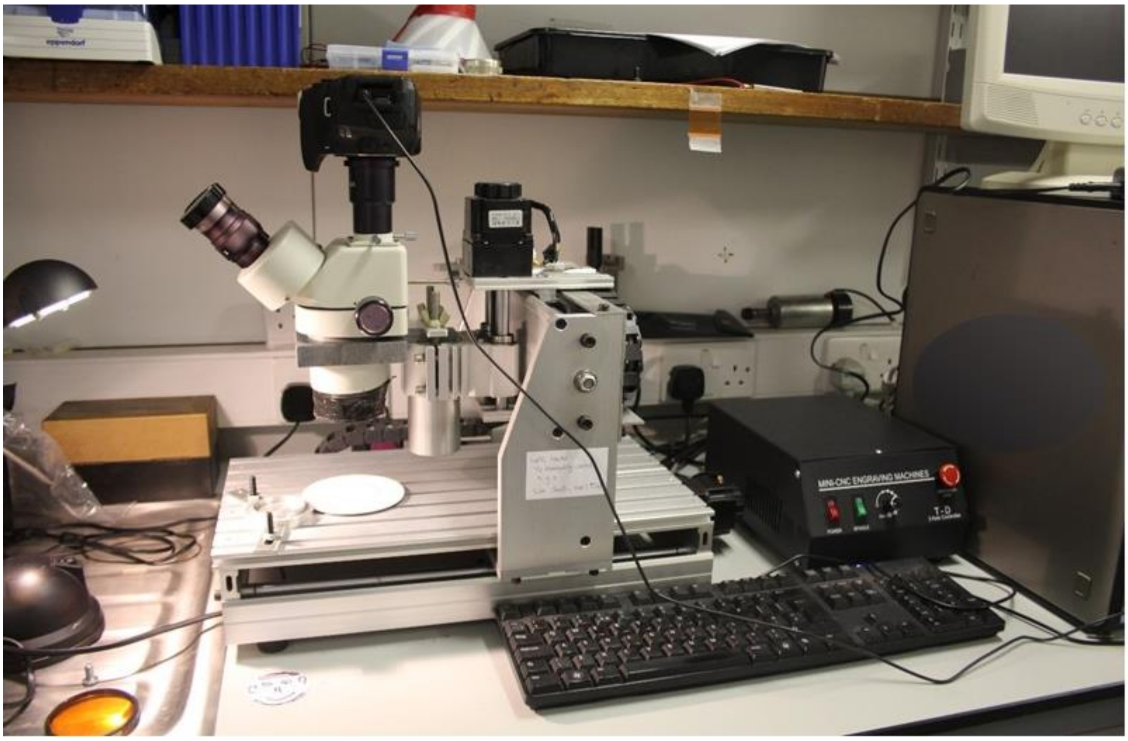

Fig SI1. Overview of the imaging rig showing all the components for white light imaging. For fluorescence work, the orange filter (left bottom) would be taped below the microscope head and the blue crimelite would be substituted for the white light.

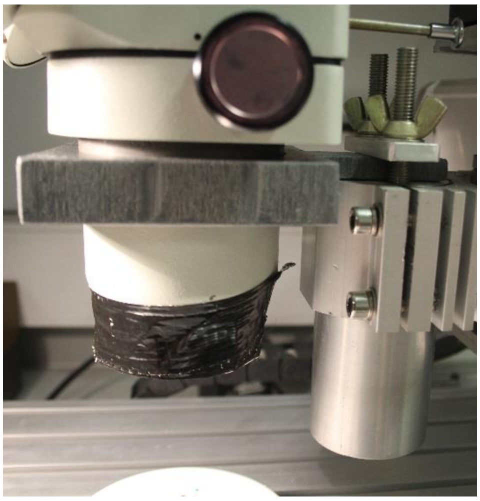

Fig SI2. Close up showing the mounting of the microscope head to the clamp that would normally house the milling motor.

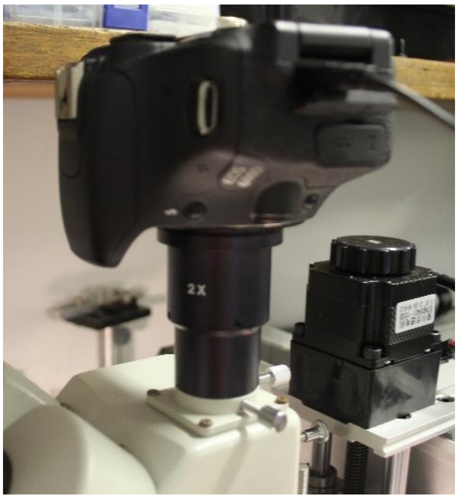

Fig SI3. The mounting of the camera to the third port of the trinocular microscope head.

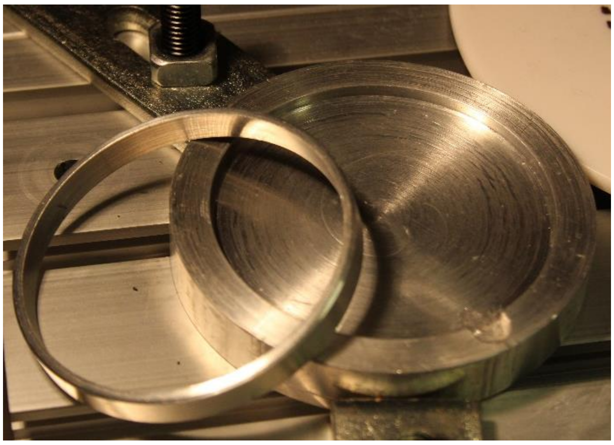

Fig SI4. Aluminium holder and weight ring machined to locate the 47 mm diameter filters correctly for automated imaging and hold them flat.

Note the notch in the edge that allows tweezers to be inserted to lift the filters in and out.

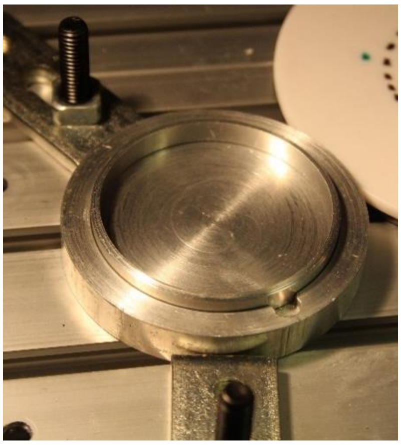

Fig SI5. The filter holder with ring in place.

2. $ZnCl_2$ solutions and densities.

Varying densities of $ZnCl_2$ solution were prepared gravimetrically from a freshly opened bottle of $ZnCl_2$ (this salt is very hygroscopic) using the weights indicated in the table. The densities were measured at 20 °C by weighing 100 mL class A volumetric flasks empty and filled with the solutions. All solutions were vacuum filtered through 0.45 µm membrane filters prior to use. They were stored in bottles/flasks with ground glass stoppers.

Table SI 1: Varying Zinc Chloride Densities.

Zinc Chloride (g)	Distilled Water (g)	Density (g/mL)
900	500	1.80
900	885	1.54
420	700	1.38
100	300	1.23
50	350	1.11

3. Separation of chitin using density differences.

Chitin-based exoskeleton material from marine organisms is quite heavily mineralised with calcium carbonate and hence has a density significantly higher than the vast majority of common plastics. This makes it relatively easy to separate such material from microplastic fragments in marine sediment samples using simple density separation. For most work we have used $ZnCl_2$ solutions with a density of about 1.35-1.38. This floats the vast majority of plastics but allows the mineralised chitin to sediment, as seen in the picture below.

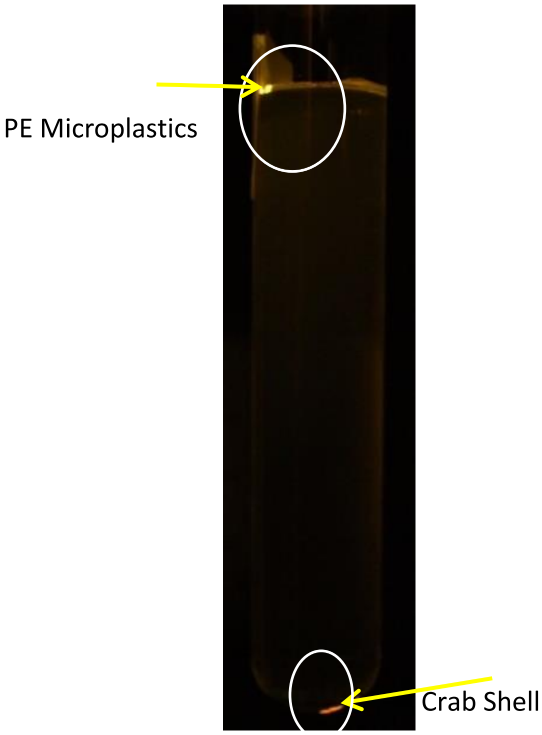

Fig SI6. Mixture of NR stained PE Microplastics and crab shell fragments suspended in $ZnCl_2$ and then centrifuged at 3900g for 10 minutes in a glass centrifuge tube. The tube was then photographed in blue light through an orange filter.

4. False positives from chitin in the ABSENCE of density separation.

NR staining of sediment samples led to the visualisation and extraction of a number of small fluorescent fragments, which were picked from filters using moistened cocktail sticks. To identify the material from which they were made, they were analysed by Raman microspectrometry using an Ar ion laser at 532 nm as source. For comparison, NR-dyed reference polymer samples were also measured. The spectra are shown below:

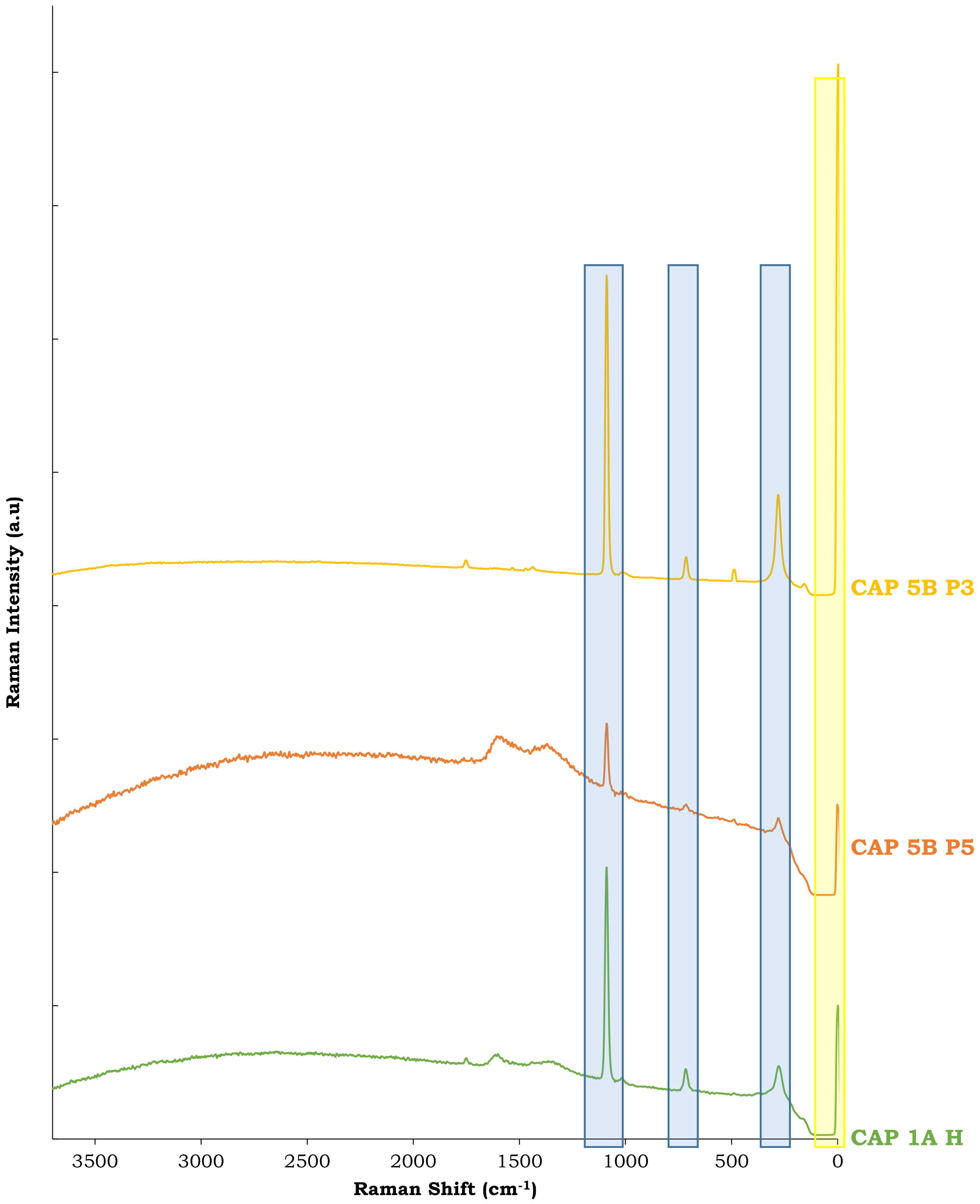

Fig. SI7: Raman **s**pectra of three fluorescent particles extracted from the non-density separated sediment samples. The band highlighted in yellow is Raleigh scattering from the laser. The bands highlighted in blue are characteristic of calcite (calcium carbonate) and are much more intense than the weak bands from the organic component.

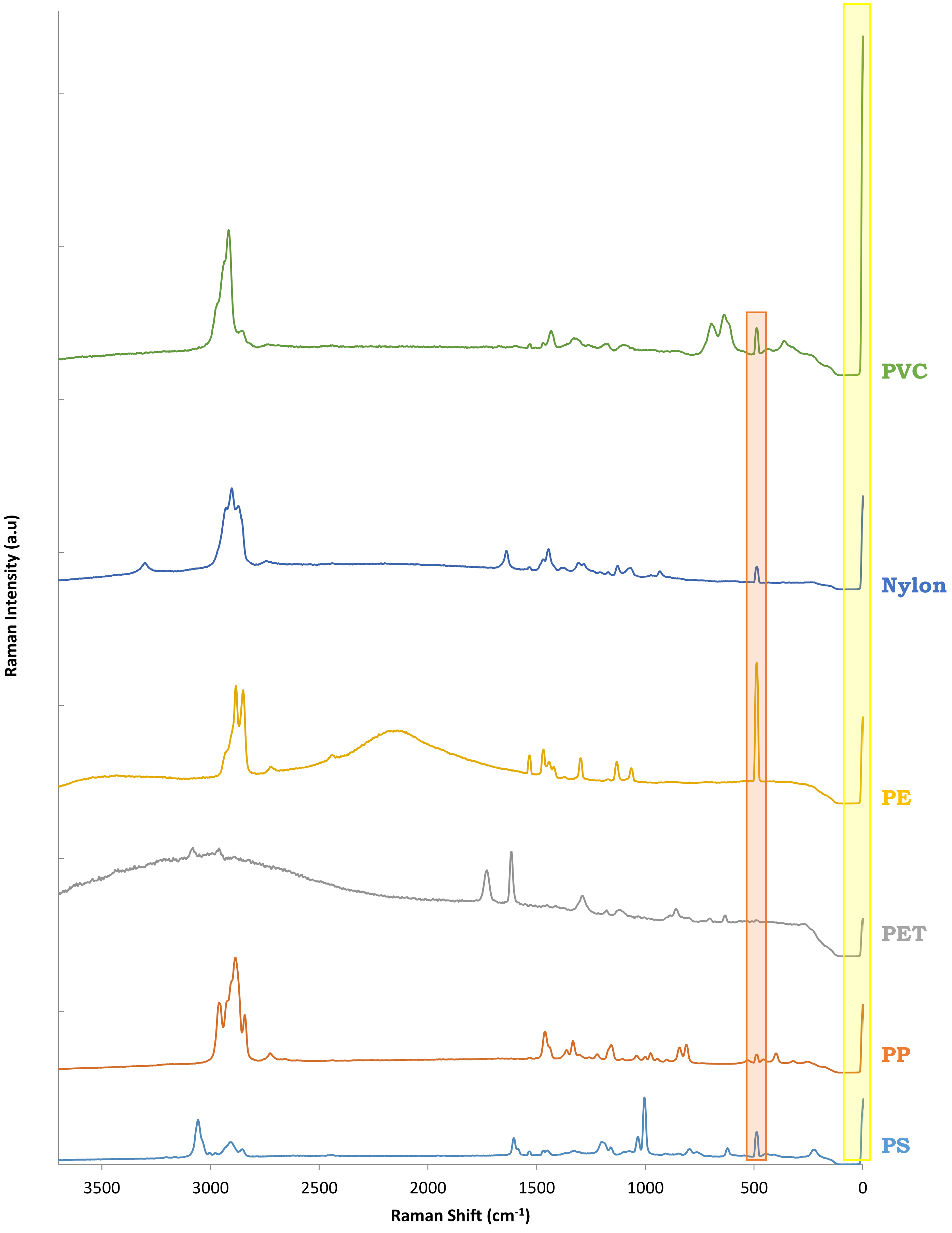

Fig SI8. Raman reference library of typical marine plastics dyed with NR imaged under identical conditions to the unknown samples. The peaks highlighted in yellow are from Rayleigh scattering and the peaks highlighted in red are background interference from the gold coated microscope slide. 532 nm laser, 100 scans, 0.5-1s integration time. The observed spectra were all consistent with literature values.

The Raman study confirmed that NR-staining fragments containing high levels of calcite could be found in samples NOT treated by density separation. After density separation, these fragments were not found, suggesting that the density separation is an important step in removing these potential false positives from the sample.

5. Solvatochromism of NR and variation of colour of dyed microplastics

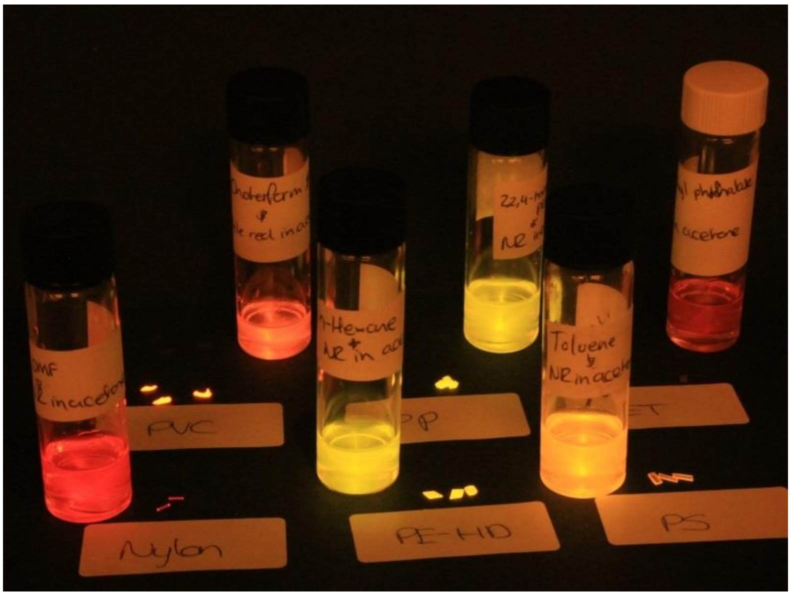

Fig SI9. Polymers dyed with Nile Red, (1000 µg mL^{-1} in acetone). Polymers are placed adjacent to vials containing solvents of similar structure/polarity. Taken under blue light (Crime Lite: 450-510 nm) through an orange filter (529 nm).

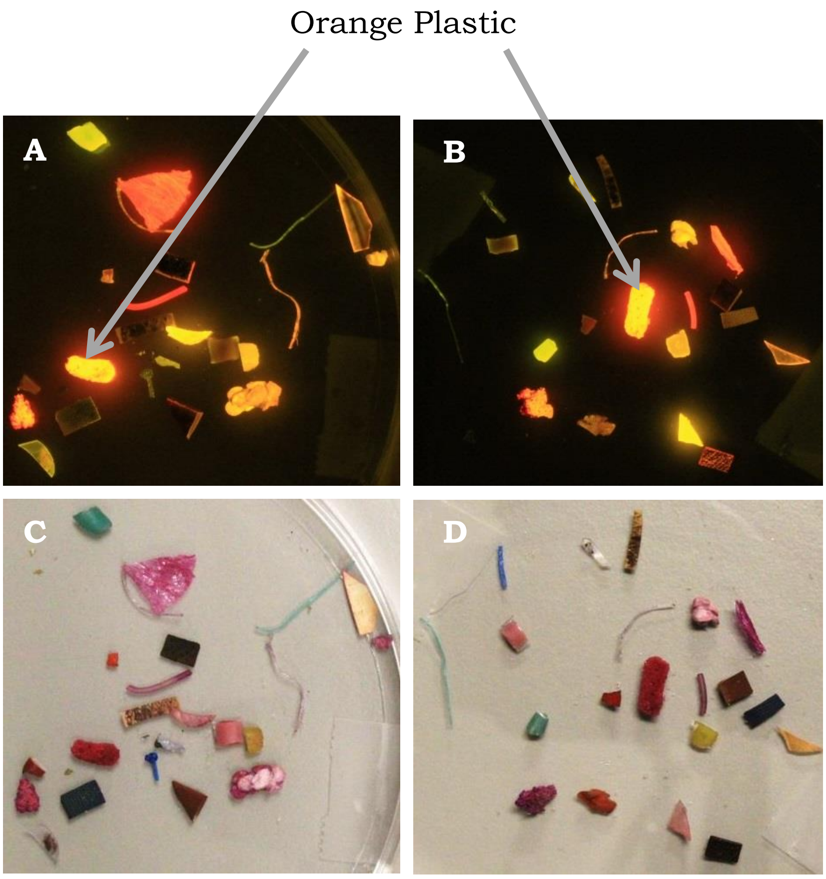

Fig SI10. Pieces of unwashed plastic waste collected from the tideline on Lowestoft beach dyed with Nile Red (91 µg mL^{-1}). A and B taken with a blue light (Crime Lite: 450-4510 nm) through an orange filter (529 nm) after 3 (**A**) and 7 days (**B**). C and D, taken under white light after 3 (**C**) and 7 (**D**) days. This demonstrates that aged/weathered plastics also stain with different colours and intensities depending on their identities.

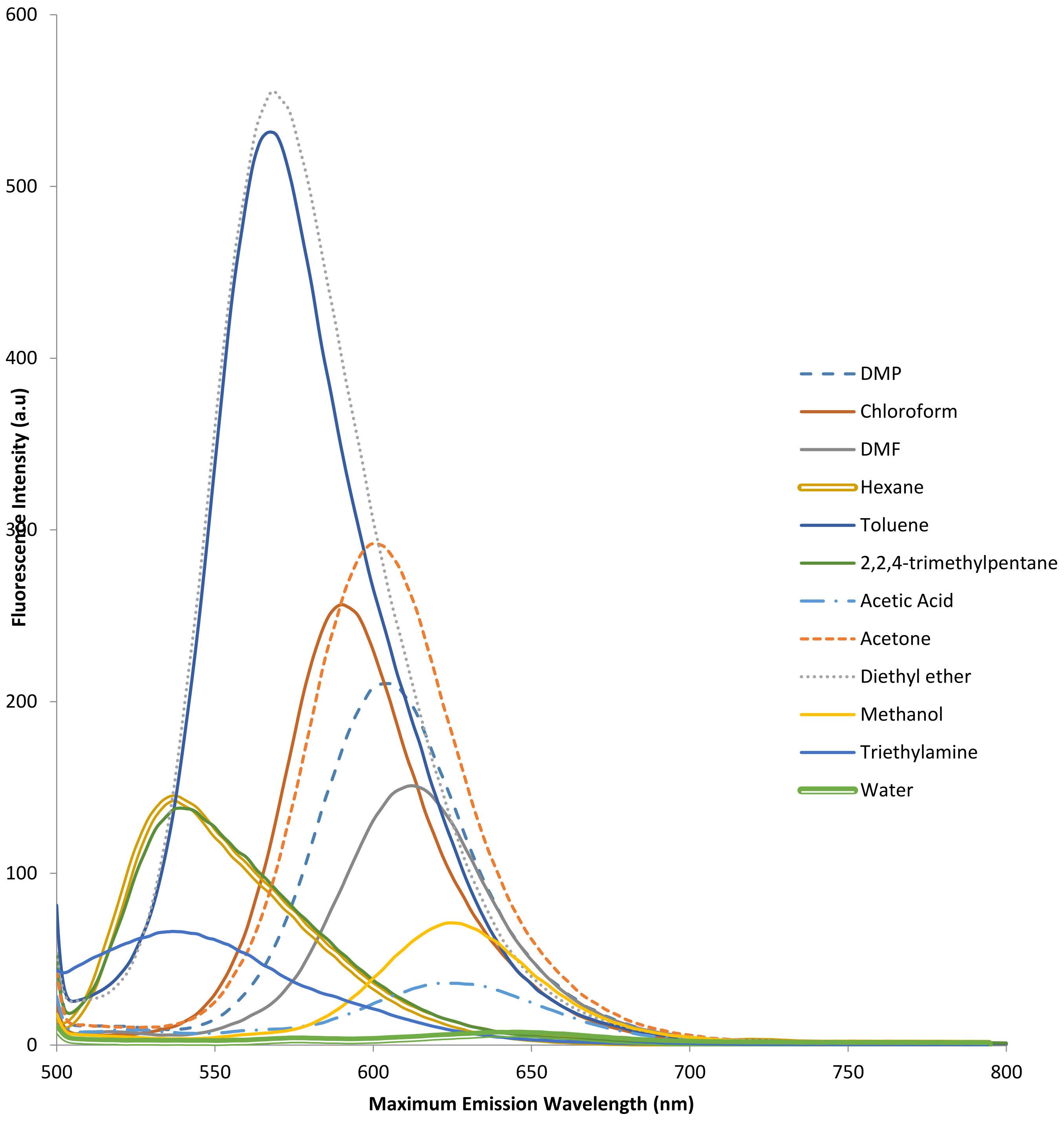

Fig SI11. Emission spectra of Nile Red fluorescence in solvents of varying polarities (mimicking the general structure of common polymers), showing the solvatochromic nature of this dye. All the spectra were measured at the same dilution, so this also gives an impression of the quantum yield (fluorescence brightness) in the different solvents, where it is strongest in toluene and almost completely quenched in water.

6. Enlarged IR spectra from the FT-IR imaging

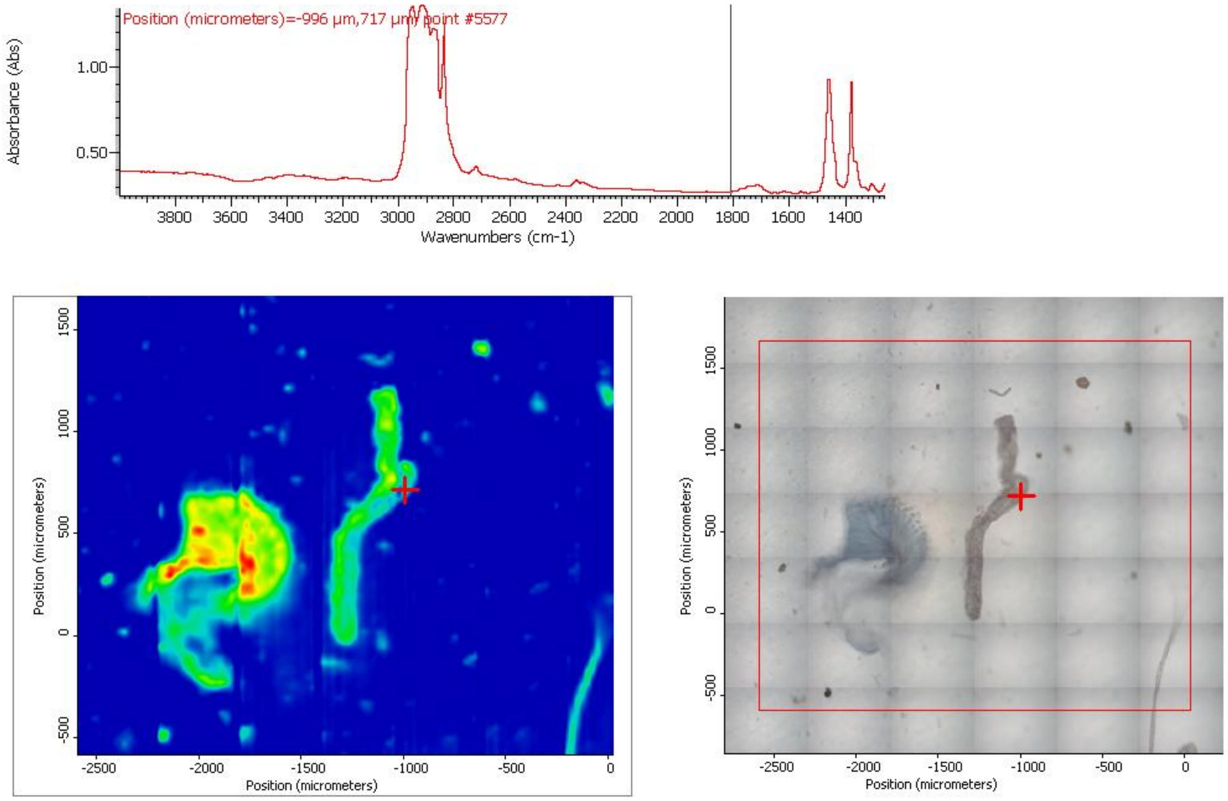

Figure SI12 Polypropylene fibre

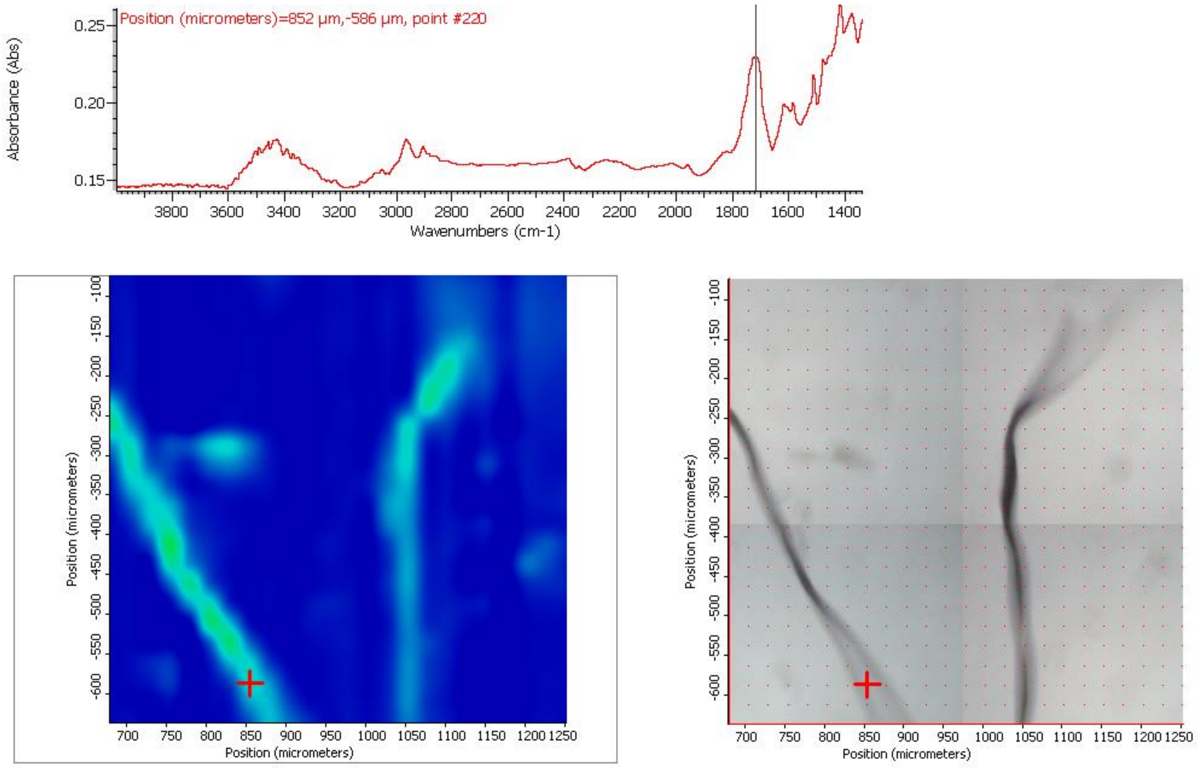

Figure SI 13 Polyester fibre

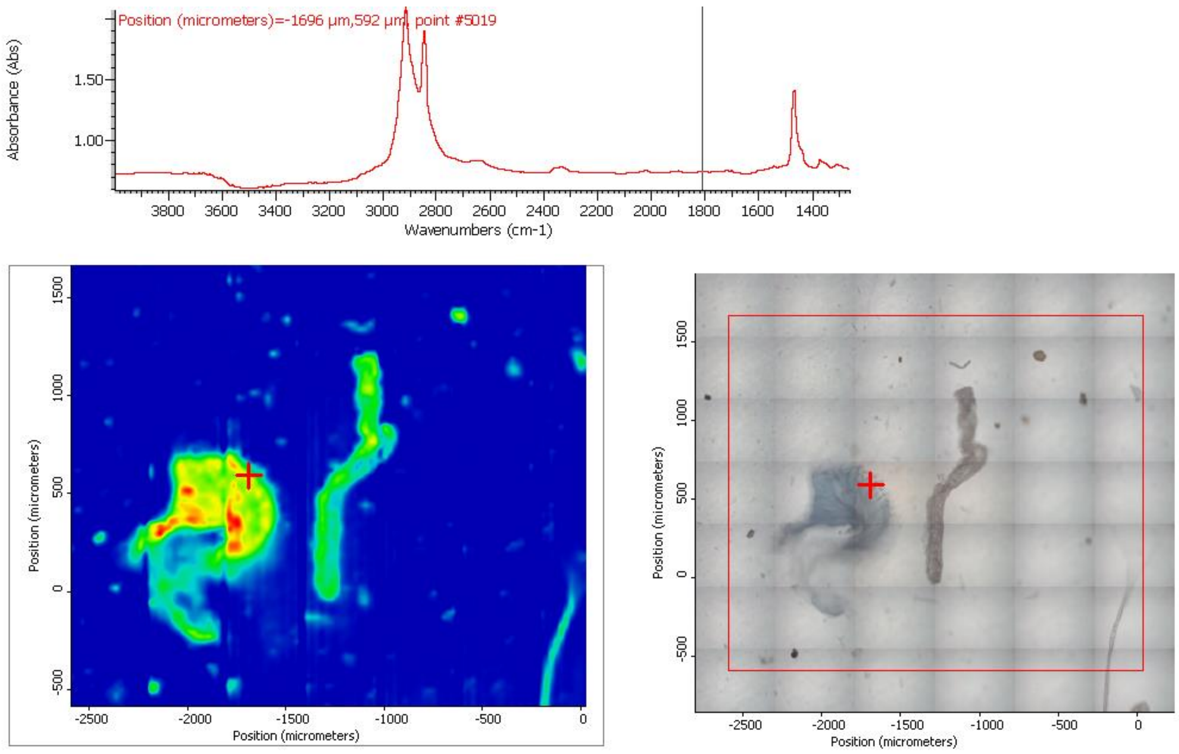

Figure SI 14 Polyethylene particle

Enlargements/details of spots identified in figure 5

Figure SI 15 - Spot 1

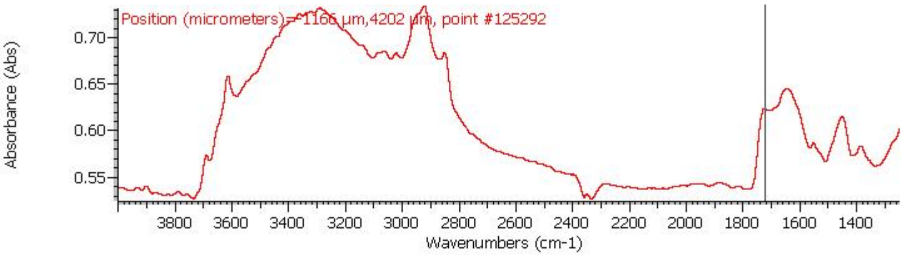

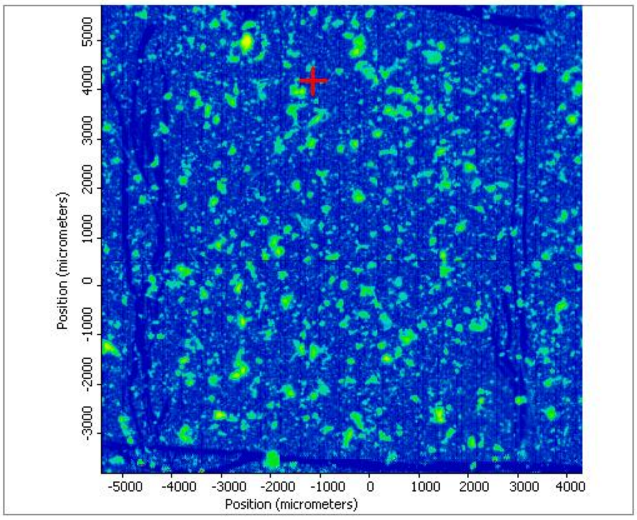

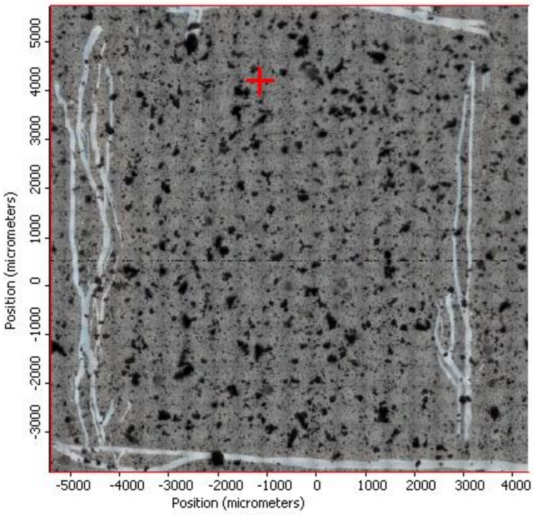

Figure SI 16 - Spot 2

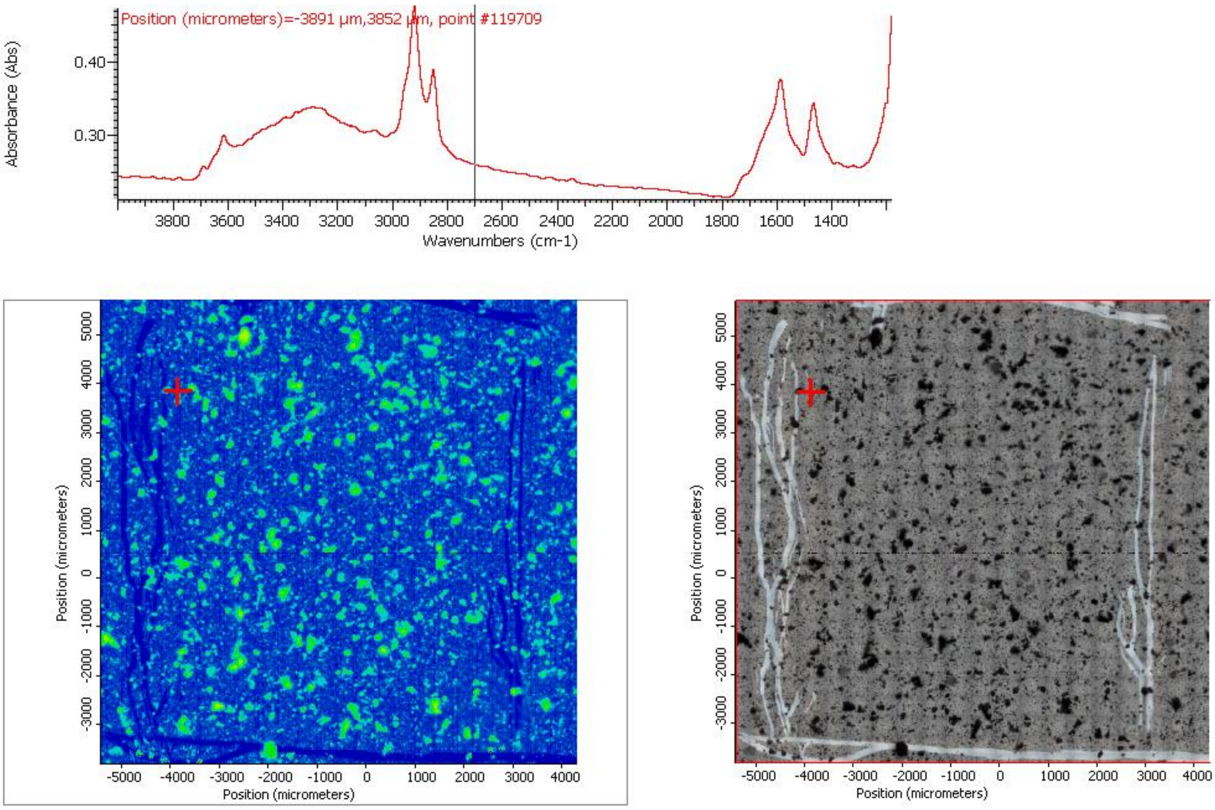

Figure SI17 - Spot 3

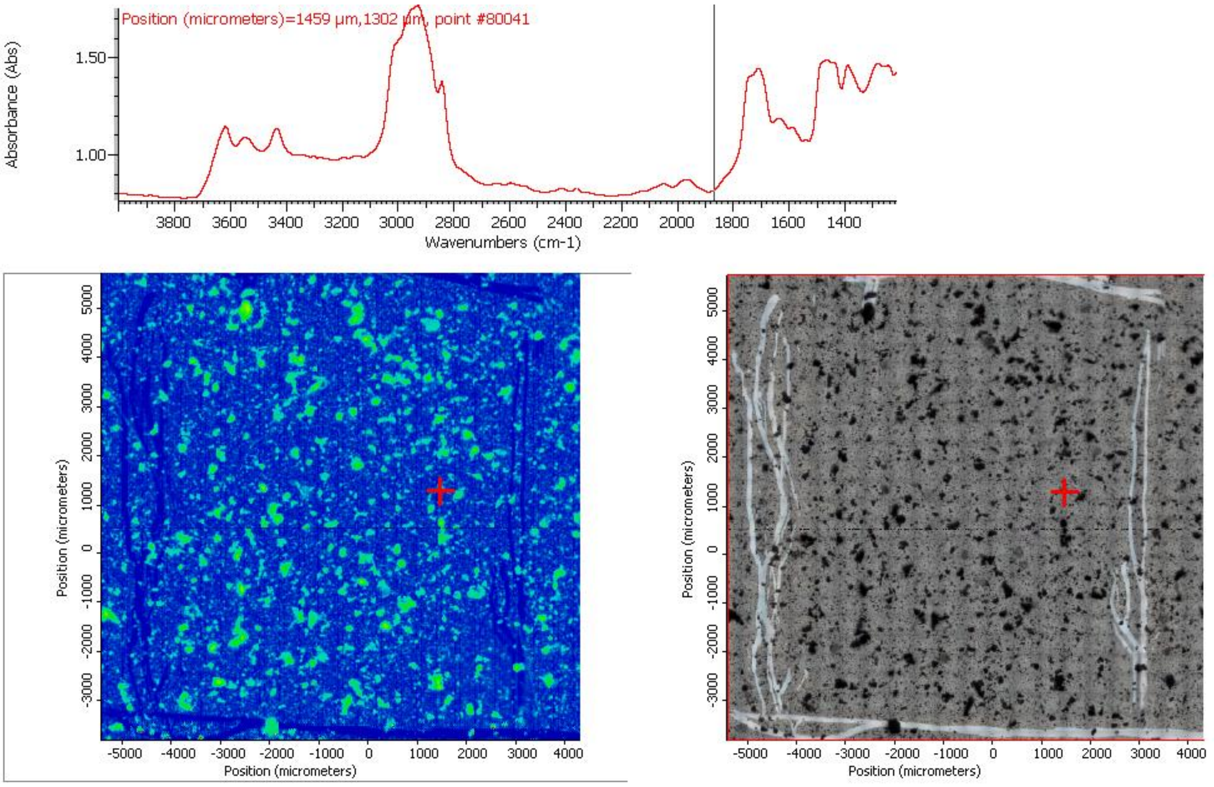

Figure SI 18 - Spot 4

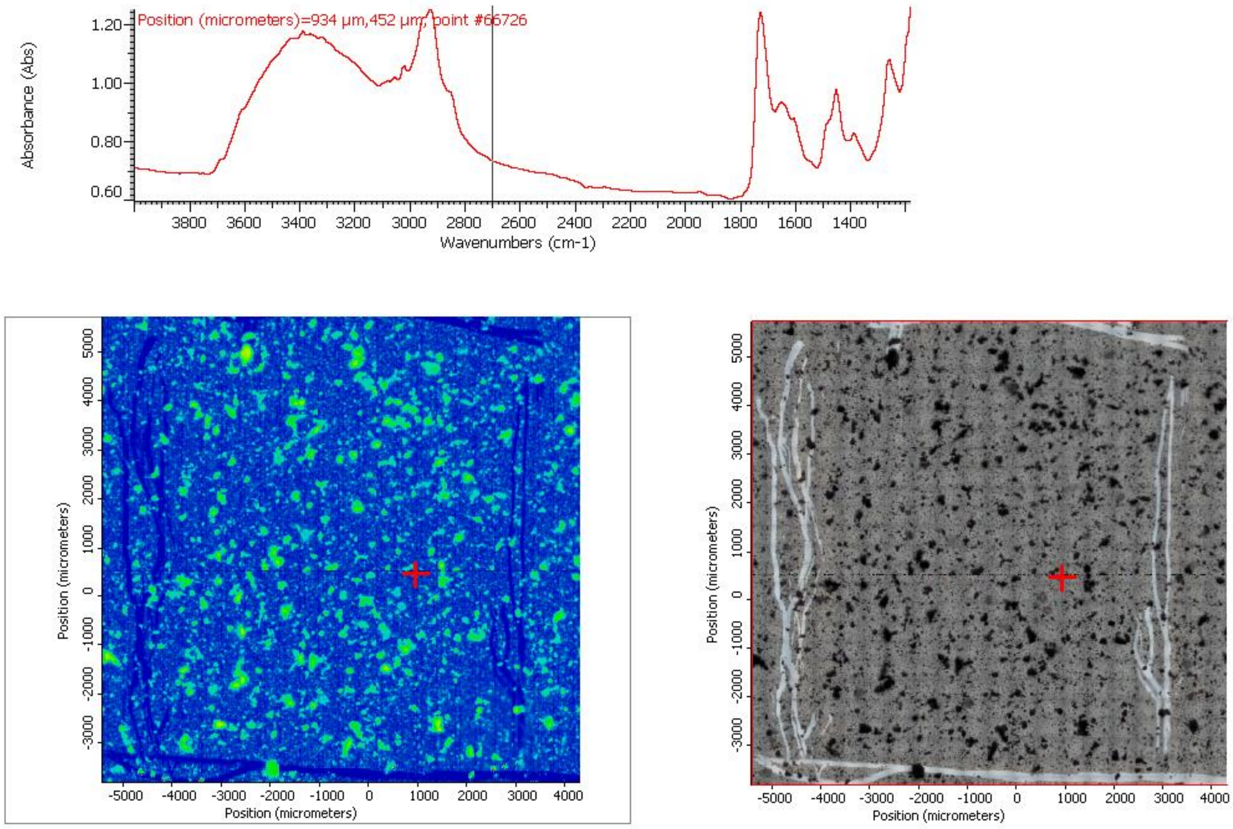

Figure SI 19 - Spot 5

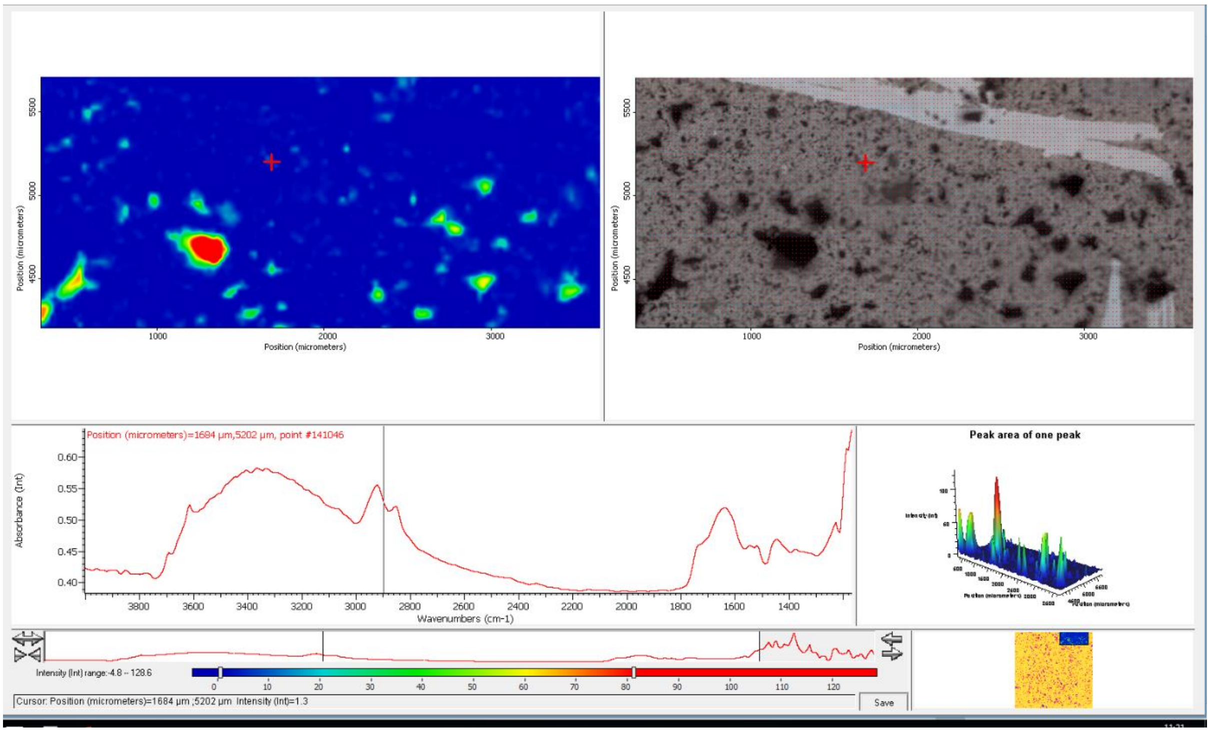

7. Fluorescence images of algae and microplastics.

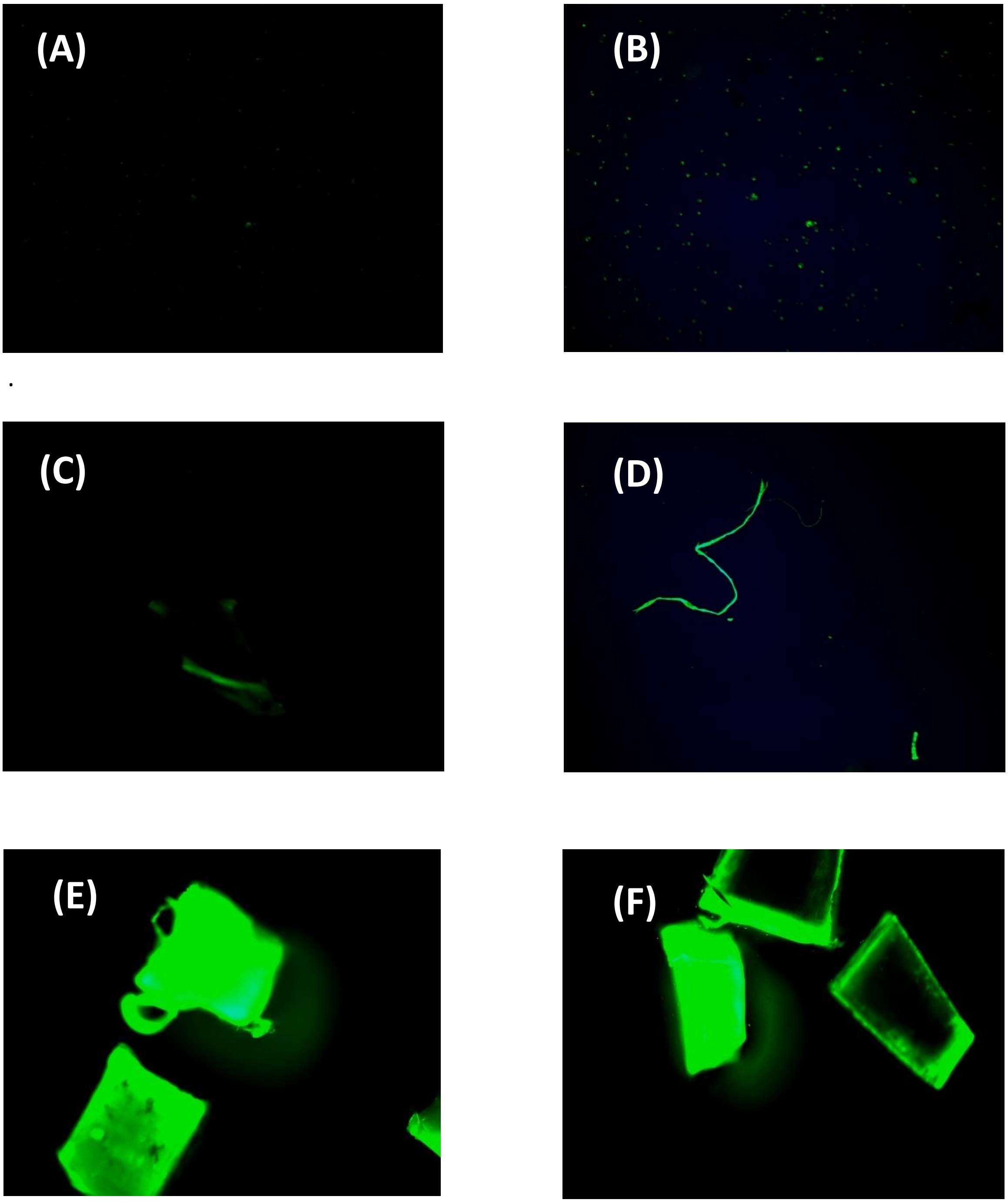

Figure SI20. Direct comparison between fluorescence of Tetraselmis and three types of microplastics (nylon, PE & PP) after NR staining and fluorescence microscopy. (A) Tetraselmis 24 s integration 80 X magnification; (B) Tetraselmis 64 s integration 80 X magnification; (C) Thick nylon fibre 4 s integration 80 X magnification; (D) Thin nylon fibre 2 64 s integration 80 X magnification; (E) PP fragment 4 s integration 80 X magnification; (F) PE fragment 4 s integration 80 X magnification.

Note: All images have the same green fluorescent colour due to the use of a 525/50 nm bandpass emission filter in this (Zeiss GFP) filter set. This is why the nylon is rather faint and needs the same exposure as the algae, since, due to the red shift of NR on the nylon surface, only the tail of the emission light is captured. The filter matches the yellow emission spectrum of PE and PP much better, hence the much brighter images. NR stained neutral lipids in algae have a yellow emission colour very similar to PE and PP, so this provides a good comparison of relative visibility under this illumination regime.

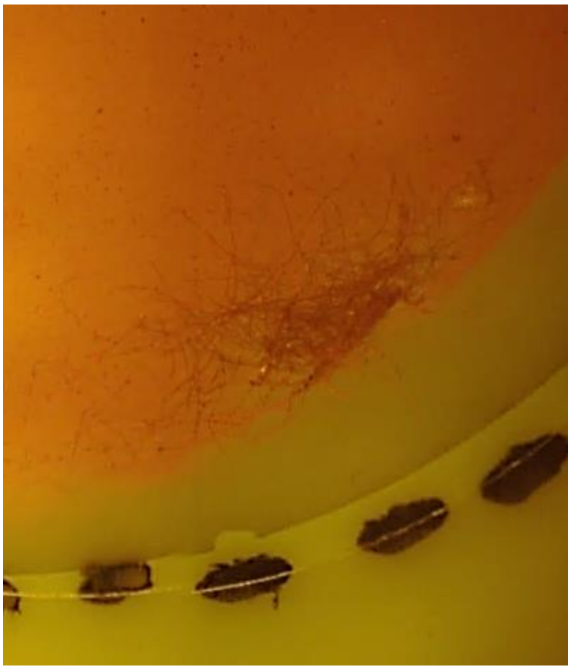

Fig SI21 Mix of 3 algae species, spiked with 20 μm nylon fibres and stained with NR, filtered and photographed using the imaging rig. Some nylon fibres can just be seen (mostly the ends of the fibres, since they act as waveguides for the emitted fluorescence and hence show bright on the ends and duller along the cylinder walls). They are entangled with a non-staining (dark) cluster of Skeletonema strands. No bright spots from Tetraselmis can be seen.

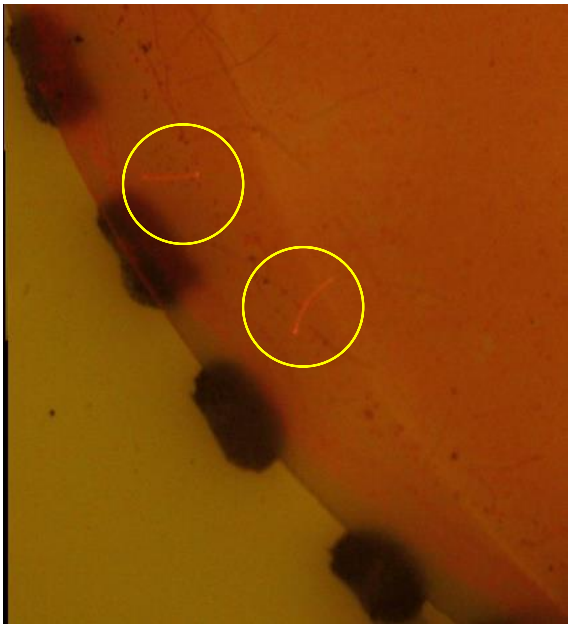

Fig SI22 Close-up showing two stained nylon fibres. Note that no stained algae can be seen even though they were present in much higher numbers.

Possible false positives from other natural organic material.

We have checked a wide range of materials in addition to the chitin and algae discussed in more detail in the text. This includes wood fragments of various types, seaweeds of different types, feathers, common whelk egg cases and a variety of different shells as well as chitin materials such as shrimp and crab claw. Most of these materials stain either very weakly (too little to show up under our imaging conditions) or not at all. The exception to this is proteinaceous material, such as the whelk egg cases and fragments of crab claw that still contained crab meat. Since proteinaceous material is biodegraded rather rapidly and is generally buoyant, we did not consider it likely that there would be significant pools of such materials in marine sediments, but for measurements of other types of samples this may need to be taken into account and additional steps added to protocols, such as treatment with protease enzymes, as is generally used for processing biota.

Carbohydrate polymers do not seem to stain. We believe that this is due to the quenching effect of hydroxyl groups and the fact that the dye does not adsorb readily to such hydrophilic surfaces. The solvatochromism data presented in ESI shows that fluorescence of NR in water and alcohols is VERY weak, compared with non-polar solvents and we believe that this is the explanation for the non-staining of typical OH-rich carbohydrates. We have tested cotton cellulose and many types of paper in other work and these materials do not stain significantly. The heavy acetylation of chitin makes it much more hydrophobic than more typical carbohydrate polymers and this is probably the explanation for its staining. Although we have not investigated other carbohydrates in detail, we have observed many spectra from non-fluorescing particles in FT-IR microscopy that closely resemble carbohydrate spectra and may have been e.g. from various seaweed carbohydrates (alginates, carageenens etc.). Many non-staining “black carbon” particles are also probably of ligno-cellulosic origin and give IR-spectral signatures consistent with this.

Wax-like materials (e.g. from cuticles of terrestrial plants) are an interesting problem, since they will have spectra very similar to polyethylene, distinguishable only by subtle differences in the CH_2 and CH_3 intensities, which will be difficult to pick up in the generally low-resolution and truncated spectra imaged on Anapore filters, hence they would very likely be mis-identified as polyethylene using current FT-IR screening methods currently in use, leading to false positives. They probably would stain (though we have not specifically checked this) and hence could also generate false positives (if present) using our staining method, hence either approach would be sub-optimal if large numbers of such fragments were present. We are not aware that this possibility has been discussed more widely in the microplastic literature, so it is probably an issue that should be further investigated, but it is beyond the scope of this proof-of-concept paper.

8 Example G-code programme for controlling milling machine motion.

```
(Generated by SmpleGCoder.com)

(XYZ in millimeters, absolute)

G00 X0 Y0

X10 Y0
```

```
G4 P5
X20 Y0
G4 P5
X30 Y0
G4 P5
X40 Y0
G4 P5
X50 Y0
G4 P5
X50 Y6
G4 P5
X40 Y6
G4 P5
X20 Y6
G4 P5
X10 Y6
G4 P5
X0 Y6
G4 P5
X0 Y12
G4 P5
X10 Y12
G4 P5
X20 Y12
G4 P5
X30 Y12
G4 P5
X40 Y12
G4 P5
```

X50 Y12

G4 P5

X50 Y18

G4 P5

X40 Y18

G4 P5

X30 Y18

G4 P5

X20 Y18

G4 P5

X10 Y18

G4 P5

X0 Y18

G4 P5

X0 Y24

G4 P5

X10 Y24

G4 P5

X20 Y24

G4 P5

X30 Y24

G4 P5

X40 Y24

G4 P5

X50 Y24

G4 P5

X50 Y30

G4 P5

X40 Y30

G4 P5

X30 Y30

G4 P5

X20 Y30

G4 P5

X10 Y30

G4 P5

X0 Y30

G4 P5

X0 Y36

G4 P5

X10 Y36

G4 P5

X20 Y36

G4 P5

X30 Y36

G4 P5

X40 Y36

G4 P5

X50 Y36

G4 P5

X50 Y42

G4 P5

X40 Y42

G4 P5

X30 Y42

G4 P5

X20 Y42

G4 P5

X10 Y42

G4 P5

X0 Y42

G4 P5

X0 Y48

G4 P5

X10 Y48

G4 P5

X20 Y48

G4 P5

X30 Y48

G4 P5

X40 Y48

G4 P5

X50 Y48

G4 P5

X0 Y0

M00
